# The implication of the crosstalk of Nrf2 with NOXs, and HMGB1 in ethanol-induced gastric ulcer: Potential protective effect is afforded by Raspberry Ketone

**DOI:** 10.1371/journal.pone.0220548

**Published:** 2019-08-12

**Authors:** Amira M. Badr, Naglaa F. EL- Orabi, Rehab A. Ali

**Affiliations:** 1 Department of Pharmacology and Toxicology, College of Pharmacy, King Saud University, Riyadh, Saudi Arabia; 2 Department of Pharmacology and Toxicology, College of Pharmacy, Ain Shams University, Heliopolis, Cairo, Egypt; 3 Department of Pharmacology and Toxicology, Faculty of Pharmacy, Suez Canal University, Ismailia, Egypt; National Institutes of Health, UNITED STATES

## Abstract

Ethanol consumption is one of the common causative agents implicated in gastric ulcer development. Oxidative stress plays a major role in the induction and development of gastric ulceration. NADPH oxidases (NOXs) and Nuclear factor erythroid 2-related factor 2 (Nrf2) are key players in ethanol-induced ulcers. High-mobility group box 1 (HMGB1), a ubiquitous nuclear protein, mediates various inflammation functions. However, the role of HMGB1 in ethanol-induced gastric ulcer is not yet elucidated. Raspberry Ketone (RK) is a natural phenolic compound with antioxidant and anti-inflammatory properties. In the present study, absolute ethanol (7.5 ml/kg) was used to induce gastric ulceration in rats. Raspberry Ketone (RK) (50 mg/kg) was given orally one hour before the administration of absolute ethanol. Interestingly, ethanol-induced gastric ulcer was associated with Nrf2 downregulation, which was correlated with NOX-1, 2 NOX-4, and HMGB1 upregulation, and was significantly reversed by RK pre-treatment. RK pre-treatment provided 80% gastroprotection. Gastroprotective properties of RK were mediated via antioxidant, anti-inflammatory (suppression of NF-kB and tumor necrosis factor-α), and antiapoptotic activities (reduction of Bax/Bcl2 ratio). Gastroprotective properties of RK were confirmed by histopathological examination. In conclusion, this study is the first to provide evidence to the role of HMGB1 in ethanol-induced gastric ulcer, and the crosstalk of Nrf2, NOXs and HMGB1. It also demonstrates that RK represents a promising gastroprotective activity comparable to omeprazole.

## 1. Introduction

Gastric ulcer is the most common gastrointestinal disorder; affecting about 10–15% of the world’s population. Although achievements and new drugs have been introduced that help to cure gastric ulcerations, the mortality is still high, and drugs still not totally effective with high side effects, that limit patients’ compliance [1]. Accordingly, authentication of the effectiveness of more effective gastroprotective agents with fewer side effects is a promising way to improve treatment outcomes.

The Pathogenesis of gastric ulceration is multifactorial and has not been completely elucidated. The surface of the gastric mucosa is protected by specific barrier mechanisms, such as bicarbonate secretion, mucus, prostaglandins, cell regeneration, and endogenous antioxidants. The imbalance between stressors; such as high gastric acid production, reactive oxygen species (ROS), inflammatory mediators, and *Helicobacter Pylori;* and protectants causes mucosal injury, and ulceration [2]. Therefore, the therapeutic strategy to protect against gastric ulcer include either intensifying gastroprotective mechanisms and/or reducing gastric-related stress factors.

The ulcerative gastric lesions are greatly associated with the use of NSAIDs and alcohol. Oxidative stress and inflammation are considered the main mechanisms of mucosal injury caused by alcohol consumption. ROS can oxidize cellular lipids, and proteins, thus, disrupting GI tract barrier and increasing gut permeability which leads to inflammation. Moreover, excess ROS stimulates macrophages, that release inflammatory cytokines such as, NF-kB, and tumor necrosis factor-α (TNF-α), thereby causing further damage to the tissue [3]. NADPH oxidases (NOXs) are enzymes acting to generate ROS; starting with superoxide anion, and ending with hydroxyl radicals [4]. Up-regulation of NOXs was found to play a role in gastric ulceration induced by aspirin, and ethanol [5].

Nuclear factor erythroid 2-related factor 2 (Nrf2) plays a significant role in the protection of the gastrointestinal tract against oxidative damage [6]. It is a transcription factor that regulates the expression of genes involved in different cellular functions. However, most attention has been directed on its ability to induce the expression of antioxidant defense proteins, including Hemoxygenase-1 (HO-1) [7]. Kovac et al. found that some NOXs are dramatically upregulated under conditions of Nrf2 deficiency and that Nrf2 partially regulates ROS by NOXs [8]. Moreover, it has been shown that Nrf2 also downregulates NFκB, thereby inhibiting proinflammatory signaling. Increased expression of Nrf2 was found to play a great protective role against gastric ulceration induced by ethanol and other insults [9,10].

High mobility group box 1 (HMGB1) proteins are abundant nuclear proteins with a wide variety of biological functions. Nuclear HMGB1 acts as chromatin structural protein, however, extracellular one acts as a proinflammatory cytokine [11]. HMGB1 was found to play a role in gastric ulceration. Nadatani et al. found that exogenous HMGB1 delays gastric ulcer healing and induces TNFα expression, on the other hand, immunoneutralization of HMGB1 or inhibiting its release promotes ulcer healing and reduces the expression of TNFα [12]. It was also reported that *Helicobacter pylori* activates HMGB1 expression, and it plays a significant role in the induction of inflammation in gastric epithelial cells [13]. However, its role in ethanol-induced gastric ulcer was not previously elucidated. Some studies visualize the role of Nrf2 activation in inhibition of HMGB1 expression in different tissues [14,15], but their crosstalk in gastric ulceration was not studied up till now.

Apoptosis also plays a role in gastric ulceration. It can be induced by excessive ROS; that activate the intrinsic apoptotic pathway; and TNF-α which is associated with extrinsic apoptotic pathway activation. The cell response (survival or death) to an apoptotic stimulus depends on the balance between pro-apoptotic proteins; Bax, Bak, and Bad; and anti-apoptotic proteins; Bcl-2 and Bcl-xl [16]. Ethanol was shown to induce apoptotic cell death of gastric mucosa which may be linked to excessive ROS and inflammation [17].

As oxidative stress, inflammation and apoptosis play a significant role in gastric ulceration pathogenesis, therefore, strong antioxidants, with additional anti-inflammatory, and/or antiapoptotic activity may represent promising candidates for gastroprotection.

Nowadays, there is a considerable emphasis about health-promoting activities of Plant-derived phenolic compounds. They are well known for their potential antioxidant, anti-inflammatory, and antiapoptotic activities [18,19]. Raspberry ketone (RK) is a natural phenolic active compound, found in blackberries, raspberries, and cranberries. It is widely used in perfumes and cosmetics industry, and The Flavor & Extract Manufacturers Association (FEMA) placed RK on ‘‘GRAS” (generally regarded as safe) status [20]. RK demonstrated protection in different animal models by decreasing lipid peroxidation and reducing inflammatory mediators such as TNF-α [21–24]. RK also showed to activate Nrf2 in a model of non-alcoholic fatty liver [23]. Thus, RK represents a promising active constituent that may protect against gastric ulceration. However, RK gastroprotective effect has never been investigated. Moreover, the effect of RK on NOXs. HMGB1 and apoptotic proteins; Bax and Bcl-2; was not previously elucidated.

Thus, the aim of this study was to determine the possible gastroprotective activity of RK against ethanol-induced gastric ulcer in rats, with comparing its effect to that of Omeprazole; an approved drug for managing gastric-ulceration. In addition, illustrating the crosstalk of Nrf2 with NOXs, and HMGB1 in ethanol-induced gastric ulcers.

## 2. Materials and methods

### 2.1. Chemicals

RK was obtained from Extrasynthese (Genay, France). Absolute ethanol, Orcinol and concentrated sulfuric acid were purchased from Sigma Aldrich Co. (St. Louis, MO, USA). All chemicals and solvents were of highest grade commercially available. Omeprazole was that available commercially (Acino, Switzerland). Primary and secondary antibodies were obtained from Abcam (Cambridge, MA, USA), and Sant Cruz (Dales, Texas, USA).

### 2.2. Animals

Adult male Wistar rats (200–220 g) were obtained from the Animal Care Center at the College of Pharmacy, King Saud University, Riyadh, Saudi Arabia. The animals were housed (5/cage) in polypropylene cages with free access to standard chow and tap water. Rats were kept at a temperature of 25±1°C under alternatively light and dark cycles and were acclimated for one week before experimentation. The study is being carried out and approved according to the ethical guidelines of the Experimental Animals Ethics Committee Acts of King Saud University (Riyadh, Saudi Arabia), with ethics refrence number of (KSU-SE-19-75) and in accordance with the ARRIVE guidelines.

### 2.3. Experimental design

Thirty rats were fasted overnight and divided randomly into five groups (n = 6). Group 1; served as a negative control; was administered vehicle only (0.05% DMSO in water; the vehicle for RK), orally. Group 2 was given vehicle one hour before giving absolute ethanol (7.5ml/kg) orally to induce gastric ulcer and acted as the positive ulcer control. Group 3, and 4 was pre-treated orally with RK at a dose of 50 mg/kg, or Omeprazole (20mg/kg) (dissolved in 0.05% DMSO in water) one hour before giving absolute ethanol (7.5ml/kg) [25,26]. Group 5 was given 50 mg/kg RK orally without ethanol. The dose of RK was selected based on a pilot experiment in our lab, in which 4 different doses of RK were examined; 25, 50, 100, and 200 mg/kg [22]. Thus, a dose of 50mg/kg was used for the experimental.

One hour after ethanol administration, the animals were anasethized using CO_2_ chamber, and then were rapidly sacrificed. The gastric contents were collected; volume was measured if applicable; and centrifuged at 5000 rpm for 15 min. Gastric juices obtained from the different treated groups were used to measure gastric juice acidity and mucin content. Stomach tissues were dissected, opened along the greater curvature and stretched on paraffin bed for macroscopic inspection. Stomach was divided into two parts, one for histopathological examination in 10% formaldehyde, and the second part of the stomach was stored at -80°C and used for the preparation of tissue homogenates, and western blot analysis. Homogenates were prepared by homogenization in phosphate buffered saline using tissue homogenizer (Omni international Inc, Kennesaw, GA, USA). Homogenates were then centrifuged at 10,000 rpm at 4°C for 15 min, and the supernatants were collected and stored at -80°C until used. Experimental methods were performed *in accordance with* the relevant guidelines and regulations.

### 2.4. Assessment of gastric mucosal injury

Ethanol-induced gastric ulcer lesions appeared as elongated bands of haemorrhagic bloody lesions. The areas of ulcerated lesions were determined using the image J analysis software (Image J, 1.46a, NIH, USA) and the percentages of the ulcerated areas relative to the total stomach area were calculated. The inhibition or gastroprotection percentage was determined according to the following formula [2]:

$$Percentage\;of\;gastroprotection\;(\%)=\frac{Ulcerated\;area\;of\;positive\;ulcer\;(Ethanol)-Ulcerated\;area\;of\;treated\;ones}{Ulcerated\;area\;of\;positive\;ulcer\;(Ethanol)}x100$$

### 2.5. Assessment of gastric juice volume, acidity, and mucus content

The stomachs were removed, opened along the greater curvature, and gastric contents (if any) were placed in tubes, and volumes were measured. The gastric contents were then centrifuged at 2000 rpm for 15 min. The Acid concentration (mEq/l) in each sample was determined by titration against 0.01 N sodium hydroxide solution, with phenolphthalein as an indicator, till reaching the endpoint [5]. Quantitative assay of the gastric mucin content was implemented according to the methodology of Al-Sayed and El-Naga (2015)[27], and was expressed as mg/ml hexoses.

### 2.6. Assessment of gastric tissue parameters

#### 2.6.1. Effect of ethanol and RK on GSH, lipid peroxidation, and antioxidant enzymes; GPx and catalase

They were assayed using specific kits (Biodiagnostic, Egypt) according to the manufacturer's instructions.

#### 2.6.2. Assessment of Nrf2 and HMGB1 expression using western blot analysis

Western blot analysis was used to determine the protein levels of Nrf2 and HMGB1 in the gastric homogenate. The samples containing 50μg of protein were mixed with an equal volume of Laemmli loading buffer. Then, the samples were denatured via incubation in thermomixer at 99°C for 5 min. Denatured samples were then separated using the vertical discontinuous sodium dodecyl sulfate polyacrylamide gel electrophoresis (SDS-PAGE) [28]. Samples were then loaded on SDS-polyacrylamide 11% resolving gel and 5% stacking gel. Gels were run in a mini-gel apparatus (Bio-Rad, Hercules, California, USA) in Tris/glycine/SDS running buffer (25 mM Tris, 192 mM glycine and 0.1% SDS, pH 8.6) at 150 V for approximately 90 min. The separated proteins were transferred to nitrocellulose membranes (Immun-Blot, Bio-Rad Laboratories, Hercules, CA, USA). Immunodetection was performed using a primary antibody: anti- Nrf2 rabbit polyclonal antibody (1:1000 dilution) Sant Cruz^®^ (Dales, Texas, USA) (C-20): sc-722, anti-HMGB1 rabbit polyclonal antibody (1:1000 dilution) [EPR3507] Abcam (Cambridge, MA, USA) (ab79823), and anti-β-actin rabbit polyclonal antibody (1:1000 dilution) Abcam (Cambridge, MA, USA) (ab8227) diluted in TBST. Blots were then incubated for one hour with secondary antibody diluted in TBST buffer. The protein bands were then visualized by Image Quant LAS 4000 mini (GE Health Care, UK) and quantified by using ImageJ version 1.45 software. The Nrf2 protein level was normalized against the loading control (β-actin) by dividing the value of the target protein by the value of the β-actin. A protein ladder was used to estimate the size of proteins. The relative value was normalized to the control whose value was fixed arbitrarily to one and assigned as a fold of induction.

#### 2.6.3. Assessment of NOX-1 and NOX-4 expression using ELISA kits

The supernatants of the stomach homogenates of different groups were evaluated for NOX-1 and NOX-4 expression using the corresponding ELISA kits according to the manufacturer's instructions. Kits were obtained from MyBioSource, USA.

#### 2.6.4. Assessment of inflammatory markers in gastric tissue

The effects of RK and ethanol on the pro-inflammatory status were assessed by measuring the levels of NF-kB, and TNF-α in the stomach homogenates supernatant obtained from different groups using ELISA Kits (Scientifics Inc, Wilmington, USA), respectively, according to the manufacturer's instructions.

#### 2.6.5. Assessment of apoptosis-related parameters

The effects of RK and ethanol on the balance of proapoptotic Bax and antiapoptotic Bcl-2 were assessed by measuring tissue levels of Bax and Bcl-2 using ELISA Kits obtained from MyBioSource, USA. The manufacturer's instructions were accurately followed. The ratio of Bax to Bcl-2 was calculated using the formula [29]:

BaxBcl−2=TissueBaxLevelTissueBcl−2Level

### 2.7. Histopathological examination

Tissue samples taken from the stomach of rats of different groups were fixed in 10% phosphate-buffered formaldehyde (pH 7.4) solution for 24 h, embedded in paraffin, cut to 5 μm-thick sections and stained with Haematoxylin & Eosin stain as well as alcian blue dye. Images were taken using light electric microscopy [30].

### 2.8. Statistical analysis

Data are presented as means ± SD. Multiple comparisons were performed using one-way ANOVA test followed by Tuckey Kramer test for post hoc analysis at P < 0.05 using GraphPad Prism version 5 software package (GraphPad Software, San Diego, CA, USA)

## 3. Results

### 3.1. Effects of RK on ethanol-induced gastric lesions

**Fig 1:**
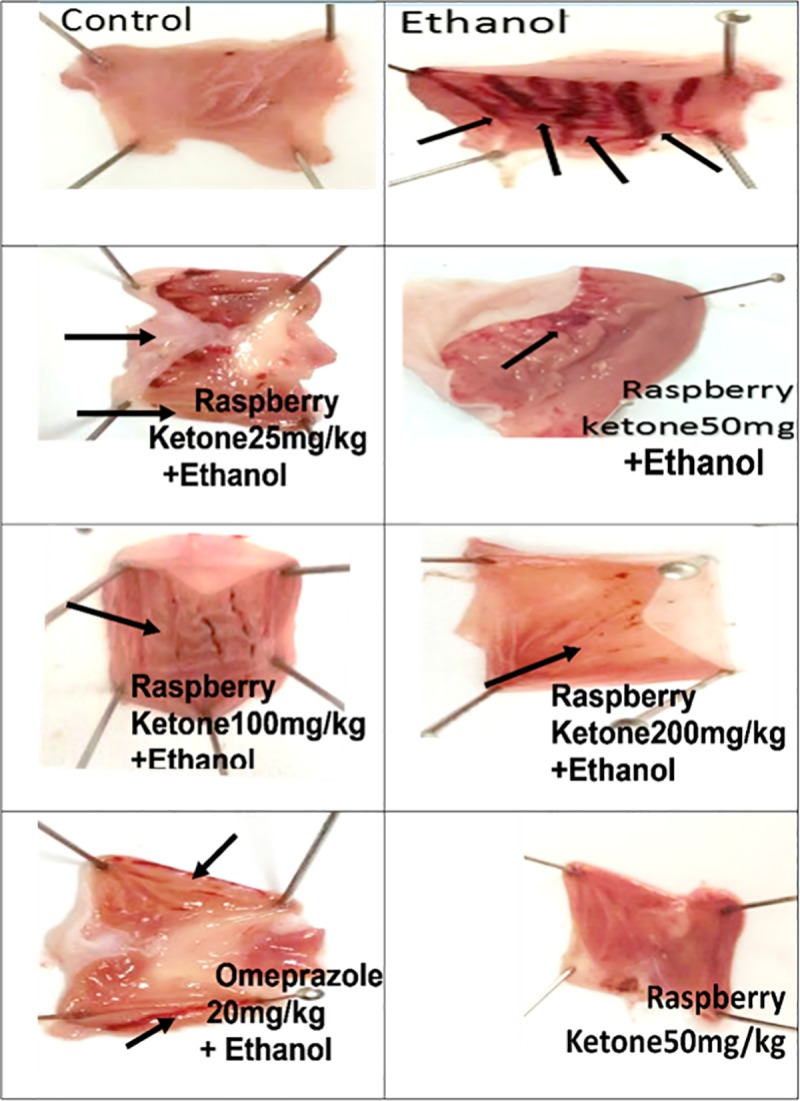
Macroscopic examination of the gastric mucosa of rats obtained from different treated groups. Control group shows no mucosal ulceration. Ethanol (Positive ulcer) group shows severe hemorrhagic lesions in the mucosa induced by the administration of 7.5ml/kg of absolute ethanol. Groups pre-treated with RK at a dose of 25, 50, 100, and 200 mg/kg or omeprazole (20mg/kg); one hour before absolute ethanol (7.5ml/kg); show marked protection against ethanol-induced gastric ulceration in rats. RK (50 mg/kg)-alone treated group shows no mucosal ulceration.

Fig 1 shows the macroscopic examination of the gastric mucosa of specimens of different groups. The normal control group, as well as the RK, only treated group showed normal gastric mucosa with no obvious ulceration. Absolute ethanol administration caused marked haemorrhagic ulcerated lesions that affecting in average 80% of the total area. Pre-treatment with RK at different doses provided a significant gastroprotection, with a dose of 50mg/kg shows a significant difference from dose of 25 mg/kg, and a non-significant difference from higher RK doses, and reduced the total ulcerated area by 80% compared to the ethanol group (positive ulcer group) (Table 1). RK 50 mg/kg was thus selected for further biochemical and histopathological assessments. Interestingly, RK (50mg/kg) showed a significant difference from omeprazole (20mg/kg) and showed a greater gastroprotective effect.

**Table 1:** Effects of different doses of Raspberry Ketone on ulcerated area % in rats.

Group	Observed Ulcerated Area %	% of gastroprotection
**Normal (-ve control)**	0	NA
**Ethanol (+ve ulcer control)**	80 ± 4.49	NA
**Ethanol+RK (25mg/kg)**	35 ± 2.4	57 ± 3.2
**Ethanol+RK (50mg/kg)**	16 ± 1.1 ^**b**^^,^ ^**c**^	80 ± 6.5 ^**b**^^,^ ^**c**^
**Ethanol+RK (100mg/kg)**	15.1 ± 1.6 ^b^^,^ ^c^	81 ± 11 ^b^^,^ ^c^
**Ethanol+RK (200mg/kg)**	11 ± 0.8 ^b^^,^ ^c^	86 ± 7.8 ^b^^,^ ^c^
**Ethanol + Omeprazole (20mg/kg)**	30 ± 2 ^b^	60 ± 7 ^b^
**RK (50mg/kg)**	0	NA

Data were shown as means ± SEM (n = 6).

NA: Not applicable

b, c: Significantly different from the Ethanol group, and from the RK (25 mg/kg) group, respectively at P <0.05

### 3.2. Effects of ethanol and RK on tissue histopathological features

**Fig 2:**
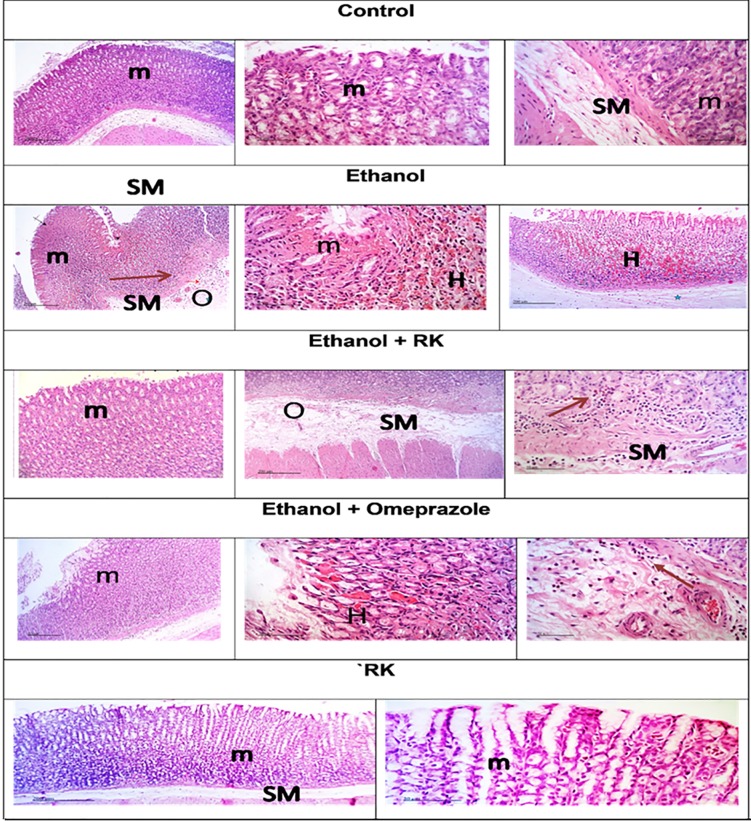
Histopathological examination of stomach sections of different treated groups. Magnification _200 and 50 μm): Sections taken from the normal control and RK-only treated group show normal histological structures of the mucosal layer (m), and the underlying submucosa (SM). Sections taken from the ethanol group show focal areas of mucosal erosions and necrotic ulceration in the mucosal layer (m), and severe hemorrhage (H), with inflammatory cells infiltration, and congested blood vessels (arrow) in the submucosal layer and edema (O). Sections taken from rats pretreated with RK (50mg/kg), one hour before ethanol, shows relatively intact glandular mucosa structures. However; some sections showed mild inflammatory cells infiltration in deeper mucosal regions and submucosal layer (arrow) with moderate submucosal edema.

Histopathological alterations in stomach autopsies of the different groups are shown in Fig 2, and Table 2. No histological alterations were observed in stomach samples taken from the negative control and RK only groups where the mucosa and the submucosa were intact. However, the stomach samples taken from rats treated with ethanol showed focal areas of mucosal erosions and necrosis with hemorrhagic regions. Extensive Submucosal edema and congested blood vessels were recorded with perivascular inflammatory cells infiltrates. In agreement with previous results, RK pre-treatment provided marked protection against ethanol-induced gastric injury; moderate infiltration of inflammatory cells in deeper mucosal regions as well as submucosal layer accompanied by moderate submucosal edema.

**Table 2:** Evaluation of histopathological changes observed in H and E stained stomach samples of different treatment groups.

Histopathological changes	Control	Ethanol	Ethanol + RK (50mg/kg)	RK (50 mg/kg)	Ethanol + Ompeprazole (20mg/kg)
Mucosal Ulceration	-	++++	++	-	++
Necrosis in the mucosal area	-	++++	+	-	+
Hemorrhage in the mucosal area	-	++++	-	-	-
Edema and inflammatory cell infiltration in the submucosa area		++++	+	-	++

++++ Severe, ++ Moderate, + Mild, and—Nil.

### 3.3. Effects of ethanol and RK on gastric juice volume, total acidity, and mucin content

**Fig 3:**
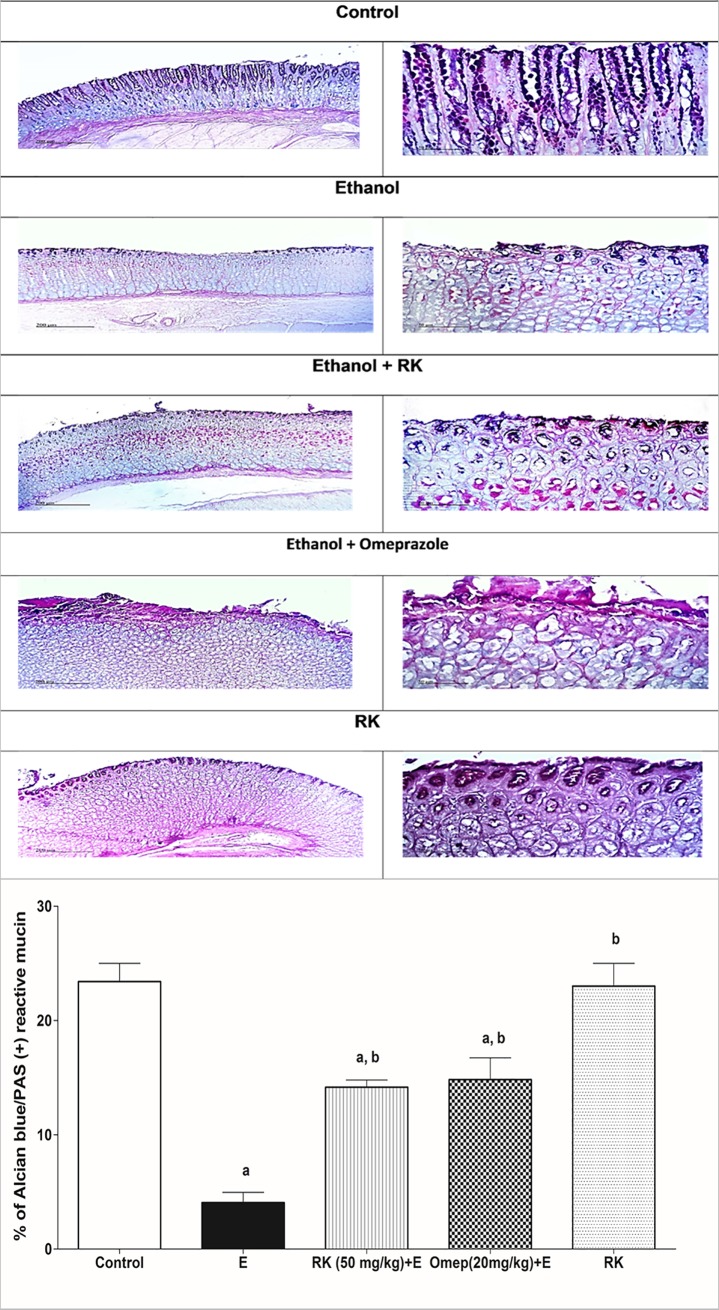
Histochemical examination of stomach sections from different treated groups using alcian blue stain to evaluate and quantify gastric mucus. Sections of the normal control group and RK (50mg/kg)-only treated group, shows a strong positive blue reaction in the intact mucosal epithelium at the tips. Section of the ethanol group shows a less positive reaction in the ulcerated mucosal layer. Section, taken from the RK (50mg/kg) pre-treated rats, show a more positive reaction compared to the ethanol group, with much more reserved mucosa. Sections of Omeprazole (20mg/kg) shows more positive reaction compared to ethanol, but less than that observed with RK. Quantitative image analysis for Alcian Blue staining expressed as a percentage of Alcian blue +ve reactive mucin, stained area averaged across 6 different fields for each rat of at least 5 rats. Data are represented as means ± SEM (n = 5). a, b: Significantly different from the control group, and ethanol group, respectively at P <0.05.

As shown in Table 3, rats pre-treated with RK showed a significant decrease in gastric juice volume, and total titrable acidity by about 60% and 50%, respectively as compared to the positive ulcer group. Moreover, RK pretreatment increased the gastric mucin content significantly by 2.7 folds as compared with the ethanol group. This finding was further supported by quantitative alcian blue histochemical staining where the ethanol group showed 80/% decrease in % staining as compared to the negative control group. However, pre-treatment with RK increased % of positive blue reaction by 2 folds as compared to the ethanol positive ulcer group (Fig 3). RK effects were comparable to omeprazole. Treatment with RK alone didn’t show any significant difference from the negative control group regarding mucin content (Table 3, and Fig 3).

**Table 3:** Effects of Raspberry Ketone on gastric juice volume, total titrable acidity, and mucin content in ethanol-induced gastric ulcer in rats.

Group	Gastric Juice Volume (ml)	Total titrable acidity (mEq/L)	Mucin Content (mg/ml hexose)
**Normal (-ve control)**	NA	NA	NA
**Ethanol (+ve ulcer control)**	1.6 ± 0.12	50 ± 0.5	1.2±0.11
**Ethanol+RK (50mg/kg)**	0.56 ± 0.06^**b**^	24±0.02 ^**b**^	3.32 ± 0.26 ^**b**^
**Ethanol + Omeprazol (20mg/kg)**	0.68 ± 0.06 ^**b**^	23 ± 0.018 ^**b**^	2.8 ± 0.23 ^**b**^
**RK (50mg/kg)**	NA	NA	NA

Data are represented as means ± SEM (n = 6).

NA: Not applicable

b: Significantly different from the Ethanol group at P <0.05

### 3.4. Effects of ethanol and RK on oxidative stress markers

As shown in Table 4, tissue levels of GSH, GPx, and catalase were significantly reduced by 60%, 68%, and 32%, respectively in the positive ulcer group as compared to the negative control group. On the other hand, RK pre-treatment normalized tissue levels of GSH and GPx and induced a significant increase in catalase enzyme level by 24% as compared to the negative control group. Moreover, RK pre-treatment significantly reduced ethanol-induced TBARs elevation by 66%. RK pre-treatment was significantly superior to Omeprazole regarding parameters. Group treated with RK alone encountered a significant elevation of GSH and catalase by 16% and 34% as compared to the negative control group.

**Table 4:** Effects of Raspberry Ketone on reduced GSH, lipid peroxides (TBARs), GPX activity, and catalase in ethanol-induced gastric ulcer in rats.

Group	Reduced GSH (umol/g Tissue)	Lipid peroxides (TBARs) (nmol/g Tissue)	GPX activity (U/g Tissue)	Catalase (U/g Tissue)
**Normal (-ve control)**	7.1 ± 0.43 ^b^	4.2 ± 0.23 ^b^	265.3 ± 18.7 ^b^	5 ± 0.16 ^b^
**Ethanol (+ve ulcer control)**	2.8 ± 0.18 ^a^	32.4 ± 2.7 ^a^	86.8 ± 5.6 ^a^	3.7 ± 0.07 ^a^
**Ethanol+RK (50mg/kg)**	7.8 ± 0.21 ^b^	11 ± 0.5 ^a^^,^^b^	262 ± 15.7^b^	6.2 ± 0.28 ^a^^,^ ^b^
**Ethanol + Omeprazol (20mg/kg)**	6.2 ± 0.4 ^b^	17 ± 0.2 ^a^^,^ ^b^	240 ± 20.2^b^	4.8 ± 0.15^b^
**RK (50mg/kg)**	8.3 ± 0.15 ^a^^,^ ^b^	4.4 ± 0.15 ^b^	298 ± 15 ^b^	6.7 ± 0.27 ^**a,**^ ^b^

Data are represented as means ± SEM (n = 6).

a, b: Significantly different from the control group, and ethanol group, respectively at P<0.05

### 3.5. Effects of ethanol and RK on Nrf2 and HMGB1 expression in gastric tissues

**Fig 4:**
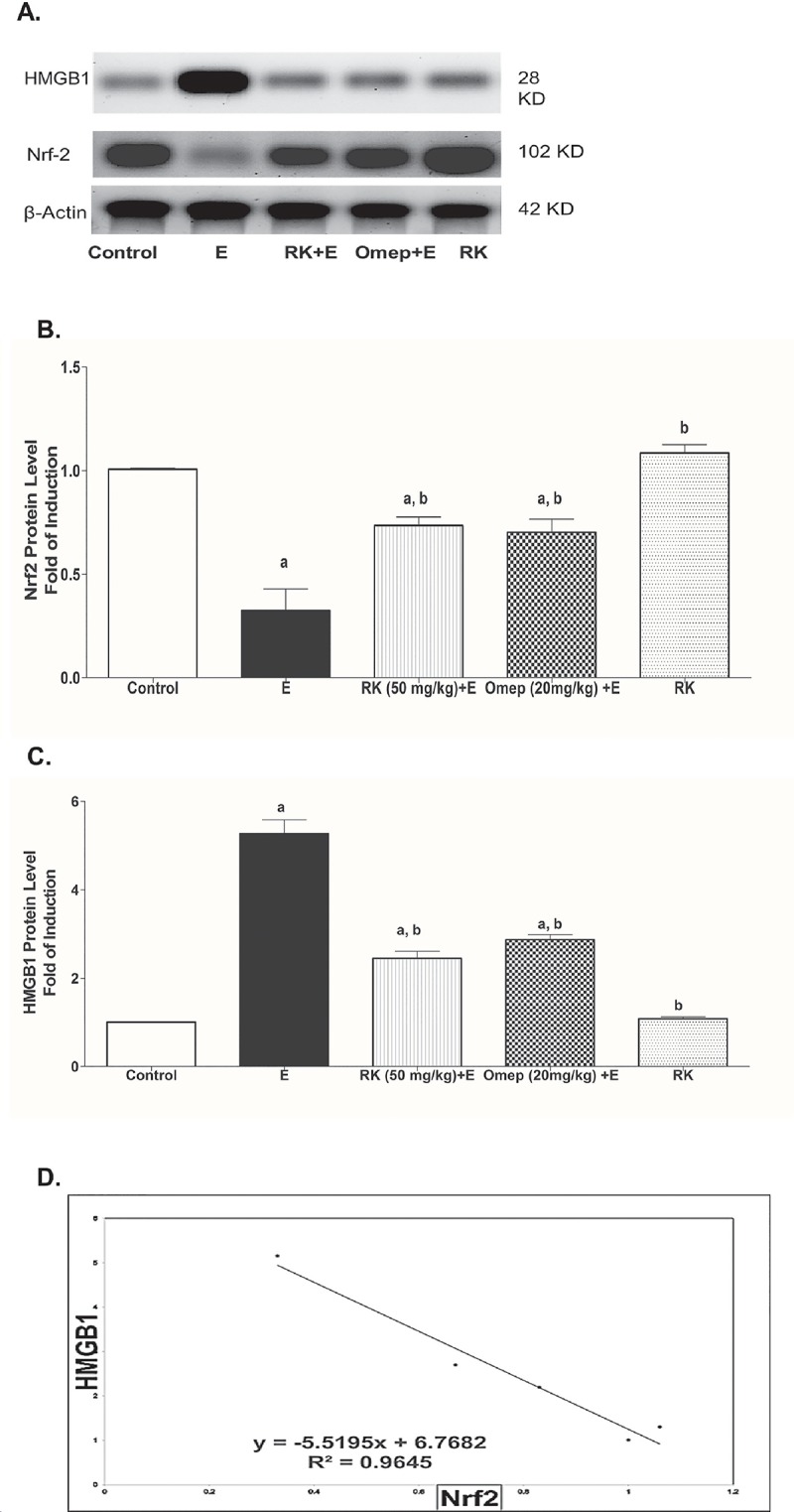
Effects of Ethanol with or without Raspberry Ketone/Omeprazole on the Gastric Nrf2 and HMGB1 protein level **(A)** Representative immunoblot of protein level from liver tissue. **Control:** normal control rats; **E:** untreated ethanol-ulcerated group; **RK+E:** Ethanol ulcerated group pretreated with Raspberry Ketone (50 mg/kg, p.o.); **Omep+E:** Ethanol ulcerated group pretreated with Omeprazole (20 mg/kg, p.o.); **RK:** Rats treated with Raspberry Ketone alone (50 mg/kg, p.o.). Sample proteins were resolved by SDS-PAGE and immunoblotted with HMGB1 antibody "upper panel", Nrf2 antibody "middle panel", and after stripping with β-actin antibody as the loading control "lower panel". The densities of immunoblots were quantified using analysis software. The sizes of the molecular mass markers expressed in kDa are indicated at the right of the panel. **(B)** Quantitative results of the immunoblots of Nrf2 (n = 3). **(C)** Quantitative results of the immunoblots of HMGB1. Protein levels are expressed as the ratio to β-actin. The relative quantities were normalized to the control and expressed as a fold of induction. Data are presented as the mean ± SEM. a, b: Significantly different from the control group, and ethanol group, respectively at P <0.05 using one way ANOVA followed by post hoc Tukey Kramer test. **(D)** Represents the correlation line and equation between Nrf2 and HMGB1 protein level.

Fig 4 shows that the tissue expression of Nrf2 was significantly reduced (Fig 4A and 4B), however that of HMGB1 was significantly induced (Fig 4C) in ethanol ulcerated group as compared to the negative control group. The two parameters showed a relatively strong inverse correlation (Fig 4D). On the other hand, pre-treatment with RK significantly increased Nrf2 tissue expression, and reduced HMGB1 protein level as compared to the ethanol ulcerated group and maintained their level not statistically significant as compared to the negative control group (Fig 4A–4C).

### 3.6. Effects of ethanol and RK on NOX-1 and NOX-4 expression in gastric tissues

**Fig 5:**
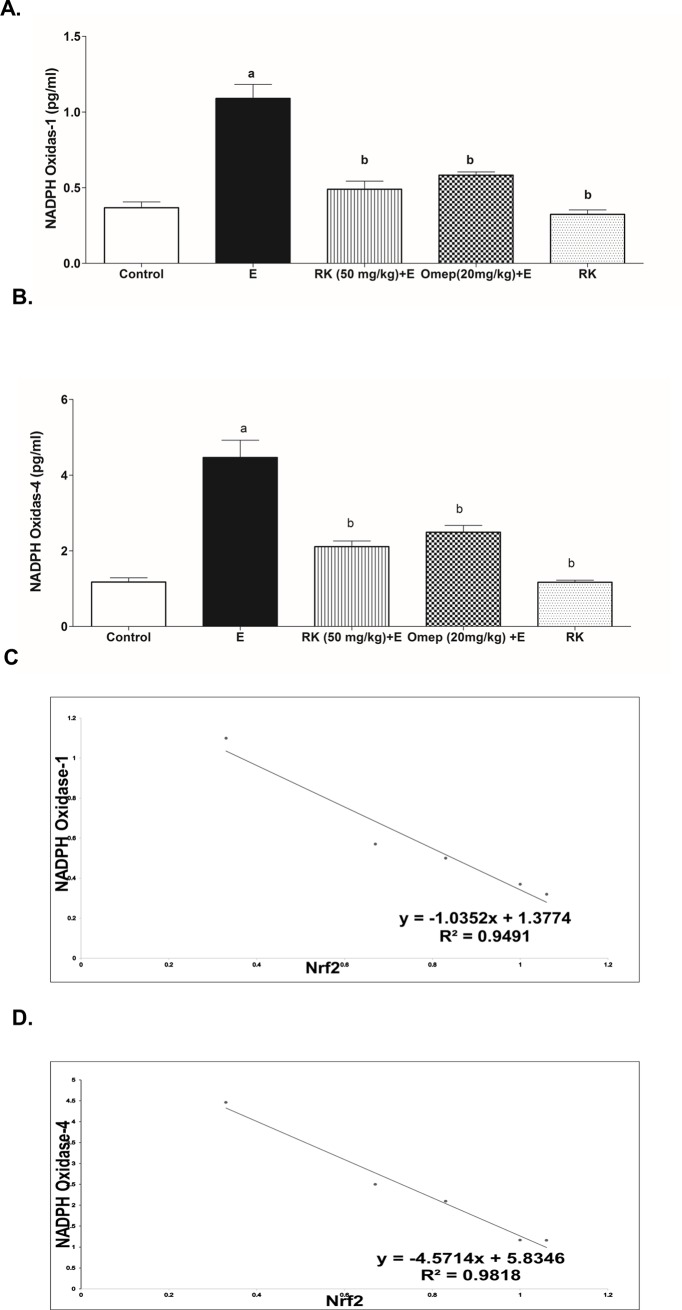
Effect of ethanol with or without Raspberry Ketone/Omeprazole on the Gastric tissue expression of **(A)** NADPH oxidase-1 (NOX-1) and **(B)** NADPH oxidase-4 (NOX-4). **Control:** normal control rats; **E:** untreated ethanol-ulcerated group; **RK+E:** Ethanol ulcerated group pretreated with Raspberry Ketone (50 mg/kg, p.o.); **Omep+E:** Ethanol ulcerated group pretreated with Omeprazole (20 mg/kg, p.o.); **RK:** Rats treated with Raspberry Ketone alone (50 mg/kg, p.o.). Data are expressed as means ± SE (n = 6). a, b: Significantly different from the negative control or Ethanol positive-ulcer group, respectively at P < 0.05, using one way ANOVA followed by post hoc Tukey Kramer test. **(C)** The correlation line and equation of Nf-2 and NOX-1 protein level. **(D)** The correlation line and equation of Nf-2 and NOX-4 protein level.

Fig 5 shows that tissue expression of NOX-1 and NOX-4 was significantly increased in ethanol ulcerated group by 3 and 4 folds respectively as compared to the negative control group (Fig 5A and 5B, respectivey). The levels of NOXs were inversely correlated to that of Nrf2, with stronger correlation calculated for NOX-4 (Fig 5C and 5D). Interestingly, pre-treatment with RK significantly reduced NOX-1 and NOX-4 tissue expression as compared to ethanol ulcerated group and maintained their level higher but not statistically significant as compared to the negative control group (Fig 5A and 5B).

### 3.7. Effects of ethanol and RK on inflammatory mediators

**Fig 6:**
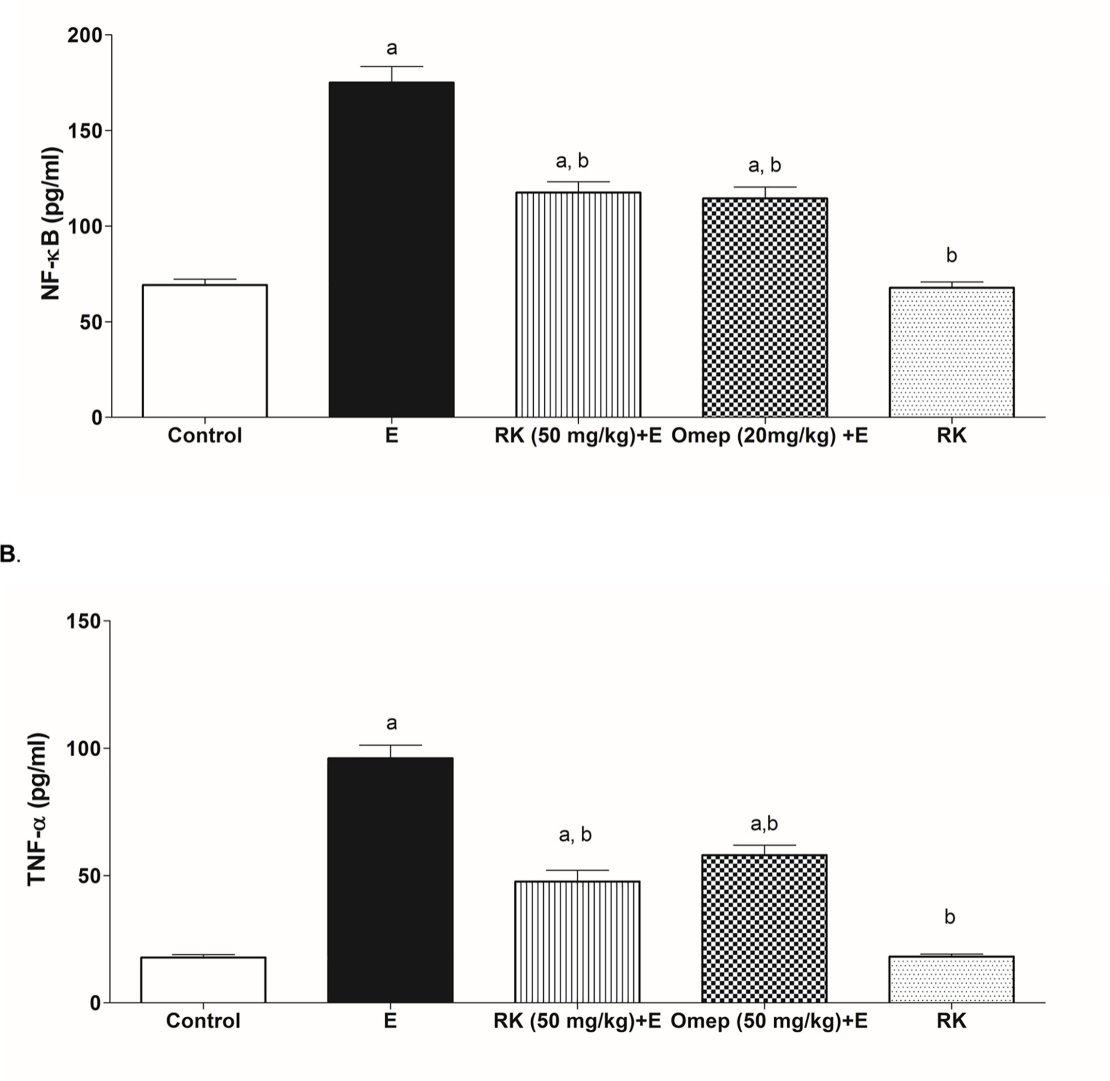
Effect of ethanol with or without Raspberry Ketone/Omeprazole on the Gastric tissue expression of **(A)** NF-kB and **(B)** TNF-α. **Control:** normal control rats; **E:** untreated ethanol-ulcerated group; **RK+E:** Ethanol ulcerated group pretreated with Raspberry Ketone (50 mg/kg, p.o.); **Omep+E:** Ethanol ulcerated group pretreated with Omeprazole (20 mg/kg, p.o.); **RK:** Rats treated with Raspberry Ketone alone (50 mg/kg, p.o.). Data are expressed as means ± SE (n = 6). a, b: Significantly different from the negative control or Ethanol positive-ulcer group, respectively at P < 0.05, using one way ANOVA followed by post hoc Tukey Kramer test.

Fig 6 shows the effects of ethanol and RK on inflammatory mediators in gastric tissues taken from the different groups. The positive ulcer group showed a marked increase in NF-kB, and TNF-α tissue levels by 2.4 and 5.3 folds respectively as compared to the control group (Fig 6A and 6B, respectively). RK at a dose of 50 mg/kg reduced NF-κB by about 35%, and TNF-α by 50% as compared to the ethanol group. RK pre-treatment effects were comparable to those of Omeprazole pre-treatment. In accordance, rats treated with RK alone did not show any significant difference as compared to the control group.

### 3.8. Effects of ethanol and RK on tissue Bax/Bcl-2 ratio

**Fig 7:**
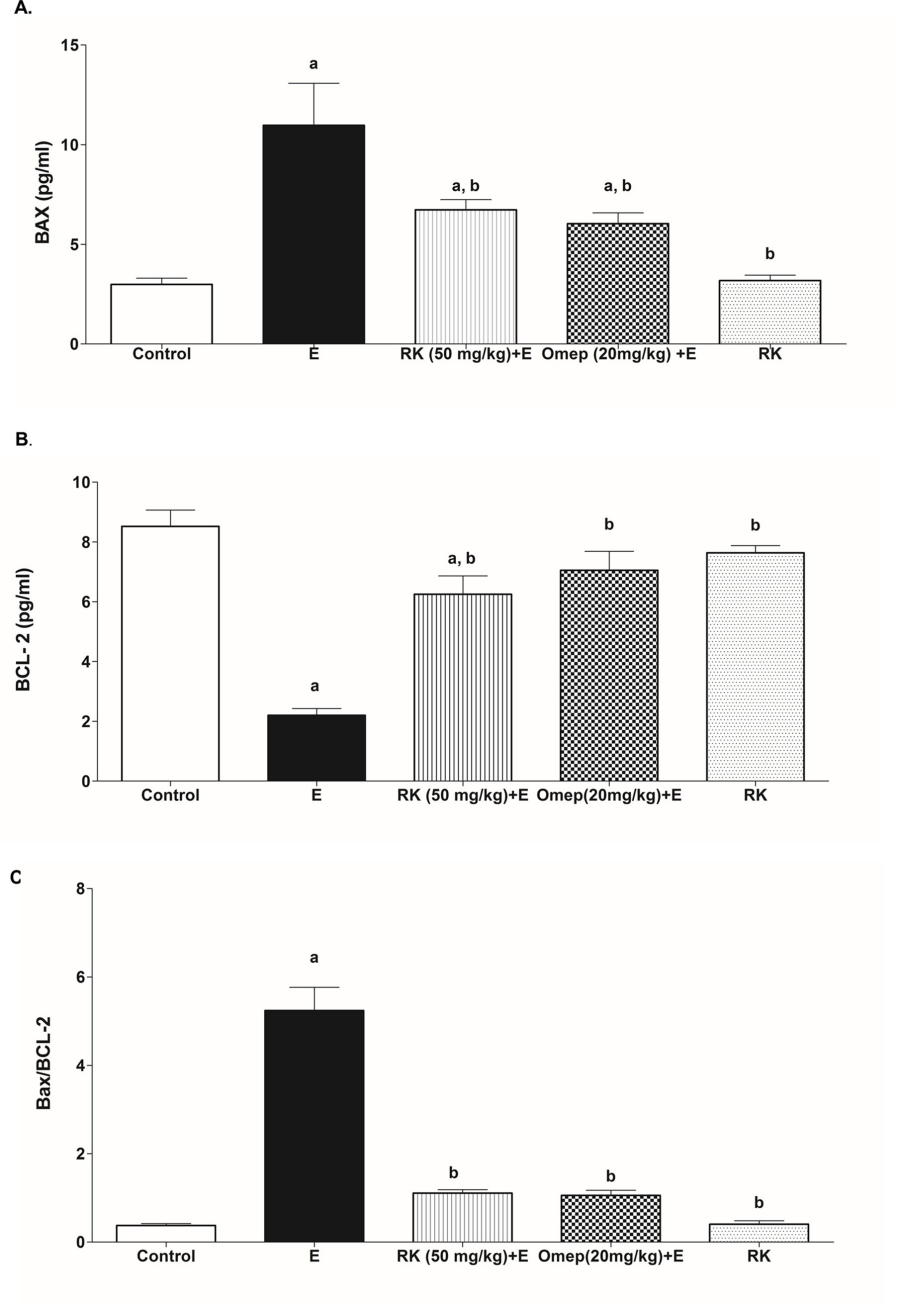
Effect of ethanol with or without Raspberry Ketone/Omeprazole on the Gastric tissue expression of **(A)** BAX and **(B)** BCL-2. **(C)** Bax/Bcl-2 expression ratio. **Control:** normal control rats; **E:** untreated ethanol-ulcerated group; **RK+E:** Ethanol ulcerated group pretreated with Raspberry Ketone (50 mg/kg, p.o.); **Omep+E:** Ethanol ulcerated group pretreated with Omeprazole (20 mg/kg, p.o.); **RK:** Rats treated with Raspberry Ketone alone (50 mg/kg, p.o.). Data are expressed as means ± SE (n = 6). a, b: Significantly different from the negative control or Ethanol positive-ulcer group, respectively at P < 0.05, using one way ANOVA followed by post hoc Tukey Kramer test.

Ethanol-induced a remarkable increase in the Bax expression, accompanied by a decrease in the expression of Bcl-2 and thus increased Bax/Bcl-2 ratio in gastric tissue by 4.4 folds compared to the negative control group. Interestingly, pre-treatment with RK resulted in a marked significant increase in Bcl-2 and reduction of Bax expression, and reduced Bax/Bcl-2 ratio significantly by 82.7% as compared to the ethanol group, and was non-significantly different from Omeprazole pre-treatment (Fig 7).

**Fig 8:**
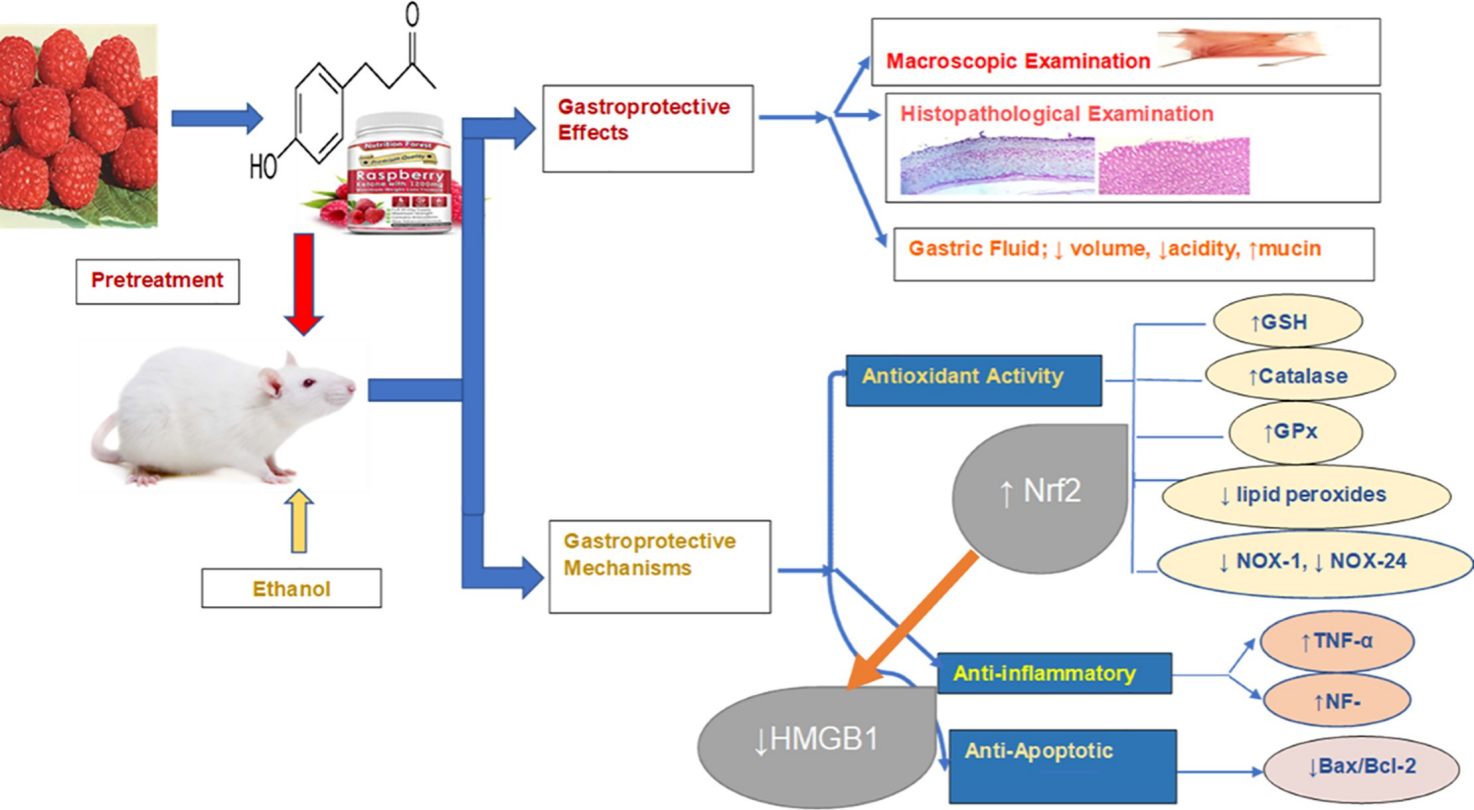
Schematic diagram of the different molecular targets involved in the gastrpprotective effects of raspberry ketone against ethanol-induced gastric ulcer in rats. Raspberry Ketone maintained ameliorated ethanol-induced oxidative stress and suppressed inflammation and apoptosis. Upregulatory effect on Nrf2, and downregulation of NOXs and HMGB1, and their crosstalk play a role in the gastroprotection afforded by RK.

Results of the current study and proposed mechanisms of RK are summarized in Fig 8.

## 4. Discussion

Although many drugs are available for the treatment of gastric ulcers, many side effects may be encountered, and failure of drug therapy may occur. Several natural plant products showed effectiveness in the treatment of gastric ulcers, such as Hesperidin and Quercetin. Some natural products are safer and have fewer side effects compared to conventional drugs [31]. RK is a phenolic compound with reported antioxidant, and anti-inflammatory activity, both activities are key players in gastroprotection [22]. However, the antiulcer activity of RK was not previously elucidated. So, the current study elucidates for the first time the possible gastroprotective activity of RK against ethanol-induced acute gastric ulceration in rats, in addition, identify the role of HMGB1 in ethanol-induced gastric ulceration, and the crosstalk of Nrf2 with HMGB1 and NOXs.

Ethanol-induced gastric ulcer model was selected in this study. The experimental model of ethanol-induced gastric ulcer represents several features of human ulcerative condition; and thus, is effective for evaluating the anti-ulcer potential of drugs along with their possible implicated mechanisms [32]. Moreover, ethanol is one of the most prevalent causes of gastric ulcer. Ethanol induces gastric injury through different mechanisms; including dehydration, which causes disruption of mucosal cellular membranes and cytotoxicity. Cytotoxicity leads to the recruitment of leukocytes that release ROS and inflammatory cytokines; both may end up in driving cells towards apoptosis. Remarkably, NF-κB plays a pivotal role in the interplay among these damaging events [33].

In the current study, ethanol-induced ulceration was observed macroscopically as severe haemorrhagic lesions in stomach specimens of rats that were administered absolute ethanol. This can be explained as ethanol causes blood stasis and disrupt gastric microvessels; which inflict hemorrhage and necrotic gastric damage [34]. These hemorrhagic lesions were further confirmed by histopathological examination using H and E, in which excessive submucosal hemorrhage was observed, associated with mucosal necrosis and ulceration, extensive submucosal ulceration, and inflammatory cell infiltration. Ethanol-induced ulceration was also associated with increased volume and acidity of gastric contents. This may be caused by the irritating effect of ethanol on the stomach, leading to the stimulation of gastric acid secretion. Moreover, ethanol administration leads to the destruction of mucus barrier, and reduced mucin level significantly as compared to control group; these were evaluated via tissue staining using Alcian -blue, followed by quantitative estimation of positive reactive mucin %. Our results are in agreement with [5,26].

Interestingly, pre-treatment of rats with RK conferred a significant gastroprotection that was confirmed by gross macroscopic examination, calculation of gastroprotection %, reduction of gastric juice volume and acidity, in addition to histopathological examination. RK also increased mucin content significantly compared to the ethanol-ulcerated group, as evident by biochemical testing, and quantitative alcian-blue staining. Actually, mucin is considered one of the major gastric defense mechanisms, and gastric acid is one of the major gastric stressors [35].Thus, RK conferred protection against ethanol damage can be mediated primarily via reducing gastric acidity and increasing protection offered by mucin.

The involvement of oxidative stress in the pathogenesis of ethanol-induced gastric injury has been confirmed by several studies. Ethanol cytotoxicity recruits inflammatory cells such as neutrophils and macrophages. A surge of ROS is being generated by activated neutrophils and macrophages, which produce O_2_^·−^, along with H_2_O_2_, HO^·^, and ONOO^−^. Ethanol also affects gastric mucosal microcirculation leading to hypoxia, which leads to extra ROS generation [36]. Ethanol-induced oxidative stress was evident in the present work by a significantly increased lipid peroxidation, paralleled with GSH depletion, and reduced antioxidant enzymes; GPx and catalase; in the ethanol ulcerated group [37]. Antioxidant activity of RK was evidenced by increasing levels of antioxidant enzymes GPx, and catalase to levels significantly higher than that of control, with a corresponding significant increase in GSH, and reduced lipid peroxidation. Antioxidant enzymes are gastric mucosa guards that scavenge free radicals and prevent their damaging effects [26]. Thus, the antioxidant activity of RK plays a great role in its gastroprotective activity.

Another important antioxidant regulatory protein that contributes to the gastroprotective machinery against excessive oxidative is Nrf2. The antioxidant response mediated by the Nrf2 pathway is considered the key defense mechanism against the oxidative stress caused by different insults. Under physiological conditions, Nrf2 is bound to its repressor Kelch-like ECH associating protein 1 (Keap1) in the cytosol [38]. Oxidative stress leads to dissociation of Nrf2 from KEAP1 in the cytoplasm, followed by its translocation into the nucleus, where it binds to antioxidant response elements and upregulates the expression of antioxidant enzymes [37]. Nrf2 induces the expression of HO-1, NAD(P)H-quinone oxidoreductase, Glutathione-S-Transferase, SOD, and proteins involved in GSH production and regeneration [8,37,39,40]. It also contributes to the maintenance of epithelial integrity that protects the mucosa of the upper GI tract against gastric luminal acidity. In the present study ethanol-induced ulceration was associated with a significantly decreased expression of Nrf2, this result is in accordance with that of Yanaka et al. and Zhang et al. [6,10]. This may contribute to ethanol-induced oxidative stress and mucosal damage. The effect of ethanol was counteracted by both RK and omeprazole, this signifies the potential antioxidant gastroprotective activity of RK, via inducing Nrf2 expression. The effect of RK on Nrf2 was previously reported in the liver [23].

Ethanol-induced gastric oxidative stress damage was recently correlated with the upregulation of NADPH oxidases (NOXs) 1 and 4 [5]. NADPH oxidases (NOXs) are a family of enzymes that are greatly involved in oxidative stress [37,41], and thus are involved in the pathogenesis of many diseases, and conditions including *H*. *pylori*-induced ulcer and lead to a further inflammatory response [4,42]. In the present study, the expression of NOX-1 and NOX-4 in gastric tissues was significantly higher in the ethanol positive ulcer group compared to the control group and thus might contribute to gastric ulcer pathogenesis. However, pre-treatment with RK caused a marked decrease in the expression of NOX-1 and NOX-4, and thus reduced ROS production and contributed to its antioxidant activity. In the present study, a strong negative correlation was found between Nrf2 and NOXs, specially NOX-4. Strong evidence suggests that NOX regulates Nrf2 activation; this has been reported for NOX4 in pulmonary epithelial cells and cardiomyocytes [43,44]. Moreover, Burtenshaw et al. reported that there are binding sites for Nrf2 located in the promoter region of Nox-4 gene [45], and Kovac et al. stated that Nrf2 deficiency is associated with NOX-2 upregulation [8]. This confirms the crosstalk of Nrf2 and NOXs evaluated in this study.

Excessive ROS can induce inflammation. The ethanol-induced inflammatory response was evident by increased gastric proinflammatory TNF-α and NF-κB in the ulcerated group. These findings are consistent with previous experiments [46]. TNF-α plays a key role in gastric inflammation via recruitment and activation of immune cells, and upregulation of NF-κB [34]. Moreover, TNF-α inhibits gastric microcirculation around the ulcerated mucosa and thus delays its healing. NF-κB also mediates critical inflammatory events in ethanol-induced gastric ulcer. It is a transcription factor that induces the expression of several proinflammatory targets including TNF-α, and IL-8 [47,48]. NF-κB is located in the cytosol of cells in an inactive state until being exposed to stress signals such as ROS and inflammatory cytokines and converted to its active form that translocates to the nucleus, interact with DNA and stimulates transcription of specific inflammatory genes. So, ROS are thus implicated in NF-κB activation, and thus induction of inflammatory response [49], thus, inhibition of the Nrf2 contributes to the inflammatory response.

Another pro-inflammatory mediator that may be involved in ethanol-induced inflammation is HMGB1. HMGB1 function differently according to its cellular location. HMGB1 translocated to the cytoplasm can mediate autophagy, however, extracellular HMGB1 can induce cytokine production via receptors for advanced glycation end products (RAGE) or toll-like receptor 4 (TLR4). So, its function can range from inflammation to repair [50]. The binding to TLR4, in particular, can lead to activation of nuclear factor κB (NF-κB) and production of TNF-α [51]. HMGB1 can be induced by different insults, including oxidative stress, autophagy, necrosis and apoptosis [19]. In the present study HMGB1 expression was induced in the ethanol-ulcerated group, and mostly contributed to inflammatory response, increased NF-kB and TNF-α. Previously, HMGB1 was found to be associated with delayed gastric ulcer healing [11], and played a role in *Helicobacter Pylori*-induced inflammation [12,52]. However, this is the first study that study the effect of HMGB1 in ethanol-induced ulceration. The effect of ethanol on HMGB1 was negatively correlated with that of Nrf2. Yu et al. showed that Nrf2/HO-1 is the main pathway for the inhibitory effect of hydrogen gas on HMGB1 in lung tissues using Nrf2-knockout mice [52]. A similar result was reported by Qu et al. who also reported increased HMGB1 expression in Nrf2 silencing. Moreover, they reported that HMGB1 inhibition is essential for Nrf2 protective activity [53].

Reduced oxidative stress associated with RK pre-treatment was also associated with reduced inflammatory mediators’ level; NF-kB and TNF-α. RK was previously reported to reduce hepatic TNF-α and inflammation in non-alcoholic steatohepatitis [21] and in isoprenaline-induced myocardial infarction [22]. Inhibition of NF-κB and TNF-α has been reported to be successful targets minimizing gastric injury [54]. RK also showed reduced HMGB1 expression that may contribute to decreased inflammatory mediators, and may be correlated to its upregulation of Nrf2. Moreover, the above findings were in harmony with the histopathological results that viewed RK-pretreated tissue samples with much less inflammatory cells infiltration, edema, and hemorrhage. This is the first study reporting effect of RK on HMGB1.

In addition to oxidative stress and inflammation, the involvement of apoptosis in the pathogenesis of ethanol-induced gastric ulceration was also described in many studies. The present results describe an imbalance of apoptotic proteins in ethanol positive ulcer group; with an upregulation of proapoptotic protein; Bax; and downregulation of antiapoptotic protein; Bcl-2; with a subsequent increase in Bax/Bcl-2 ratio as compared to the negative control. TNF-α and ROS are both implicated in the activation of apoptotic pathways. TNF-α can bind to TNFR-1 (death receptor), that stimulates extrinsic apoptotic pathways [48]. ROS can activate the mitochondrial apoptotic pathway. Mitochondrial apoptotic pathway is initiated by pro-apoptotic signalling protein such as Bax, which stimulates cytochrome c release, with subsequent caspases activation. Apoptosis can be inhibited by antiapoptotic proteins such as Bcl-2. Bcl-2 modulates the apoptotic pathway by binding and neutralizing the mitochondrial proapoptotic proteins [55]. The ratio between pro- and antiapoptotic proteins thus control cell destiny. Apoptosis can lead to DNA fragmentation that allows the release of HMGB1 to the cytoplasm and extracellular spaces and contributes to further tissue damage [55,56].

The current data revealed that RK corrected the imbalance between pro- and antiapoptotic proteins caused by ethanol administration. RK reduced Bax/Bcl-2 ratio to levels comparable to control. Effect of RK on apoptosis was not previously studied. The antiapoptotic activity can be explained by RK ability to suppress ROS and TNF-α; both have been reported to enhance gastric apoptosis [49].

Taken together, this study is the first to highlight the role of HMGB1 in ethanol-induced gastric ulcer, and correlate it to Nrf-2. It also provides scientific evidence of the potential gastroprotective activity of RK. RK anti-ulcer activity was compared to that of omeprazole as a positive control. RK was superior to Omeprazole in % of gastroprotection and antioxidant activity-related parameters. Regarding other parameters, RK was comparable to Omeprazole. Mechanisms of RK activity include antioxidant effect which may be caused by Nrf2 upregulation, anti-inflammatory activity associated with reduced HMGB1 expression, and antiapoptotic activity (Fig 8). This supports the importance of RK as a promising natural anti-ulcerative supplement. Further studies are needed to investigate RK efficacy in other gastric ulcer models and clinically as an alternative or adjunct for the management of gastric ulcers. Further mechanisms may also be explored.

## Supporting information

S1 Fig Western blot of Nrf2. (JPG)

S2 Fig Western blot of HMGB1. (JPG)

S3 Fig Western blot of β-actin. (JPG)

## Abbreviations

Fig: Figure
GPx: Glutathione Peroxidase
GSH: Reduced Glutathione
HMGB1: High-mobility group box 1
NF-kB: Nuclear Factor-kB
NOX: NADPH Oxidase
Nrf2: Nuclear factor erythroid 2-related factor 2
RK: Raspberry Ketone
ROS: Reactive oxygen species
TNF-α: Tumor necrosis factor- α
