# Supplementary figures and images for: The implication of the crosstalk of Nrf2 with NOXs, and HMGB1 in ethanol-induced gastric ulcer: Potential protective effect is afforded by Raspberry Ketone
Source: PLoS One. 2019 Aug 12;14(8):e0220548. doi: 10.1371/journal.pone.0220548 (PMC6690542; doi:10.1371/journal.pone.0220548)

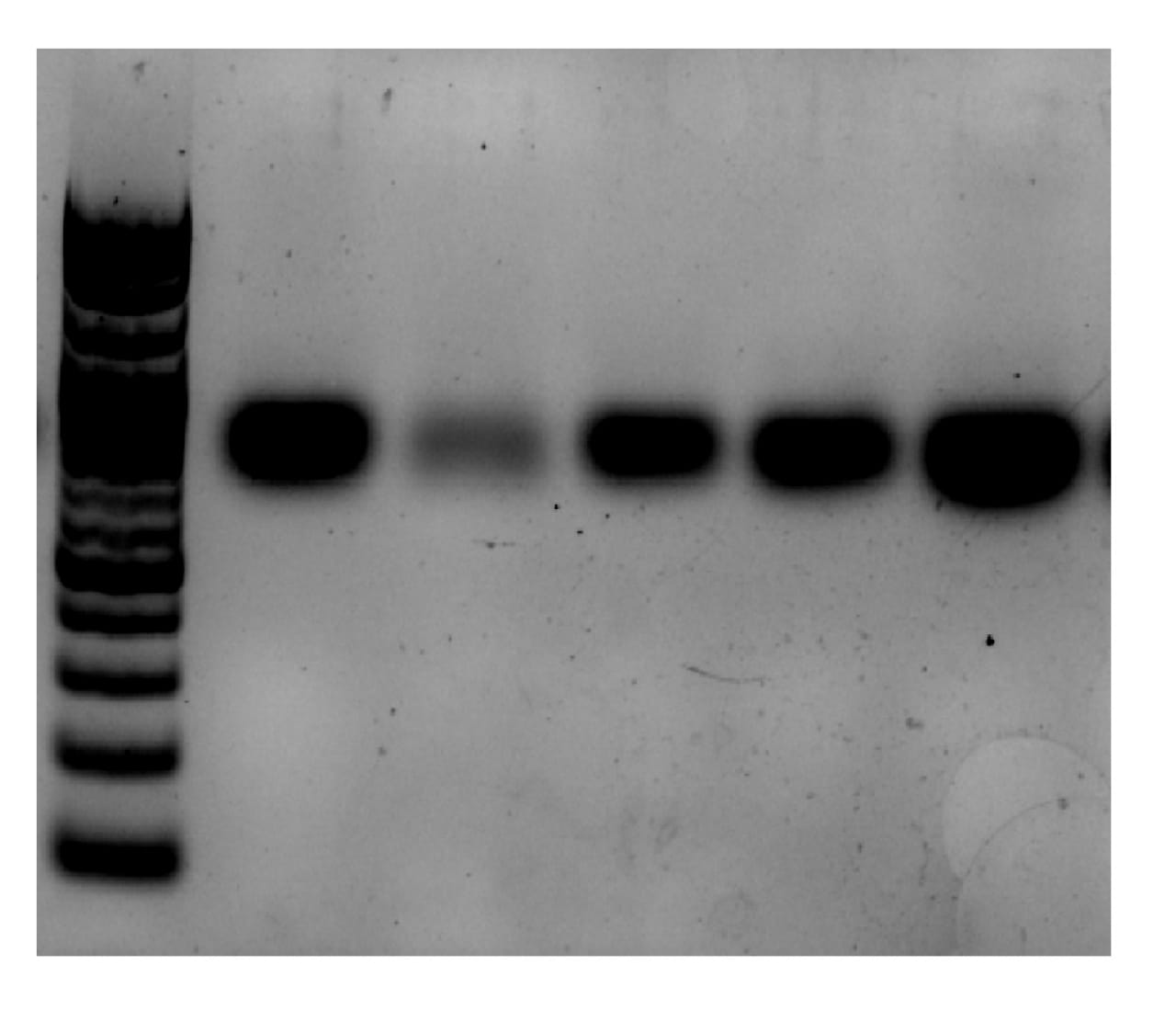

Supplement: S1 Fig — (JPG) [file pone.0220548.s001.jpg]

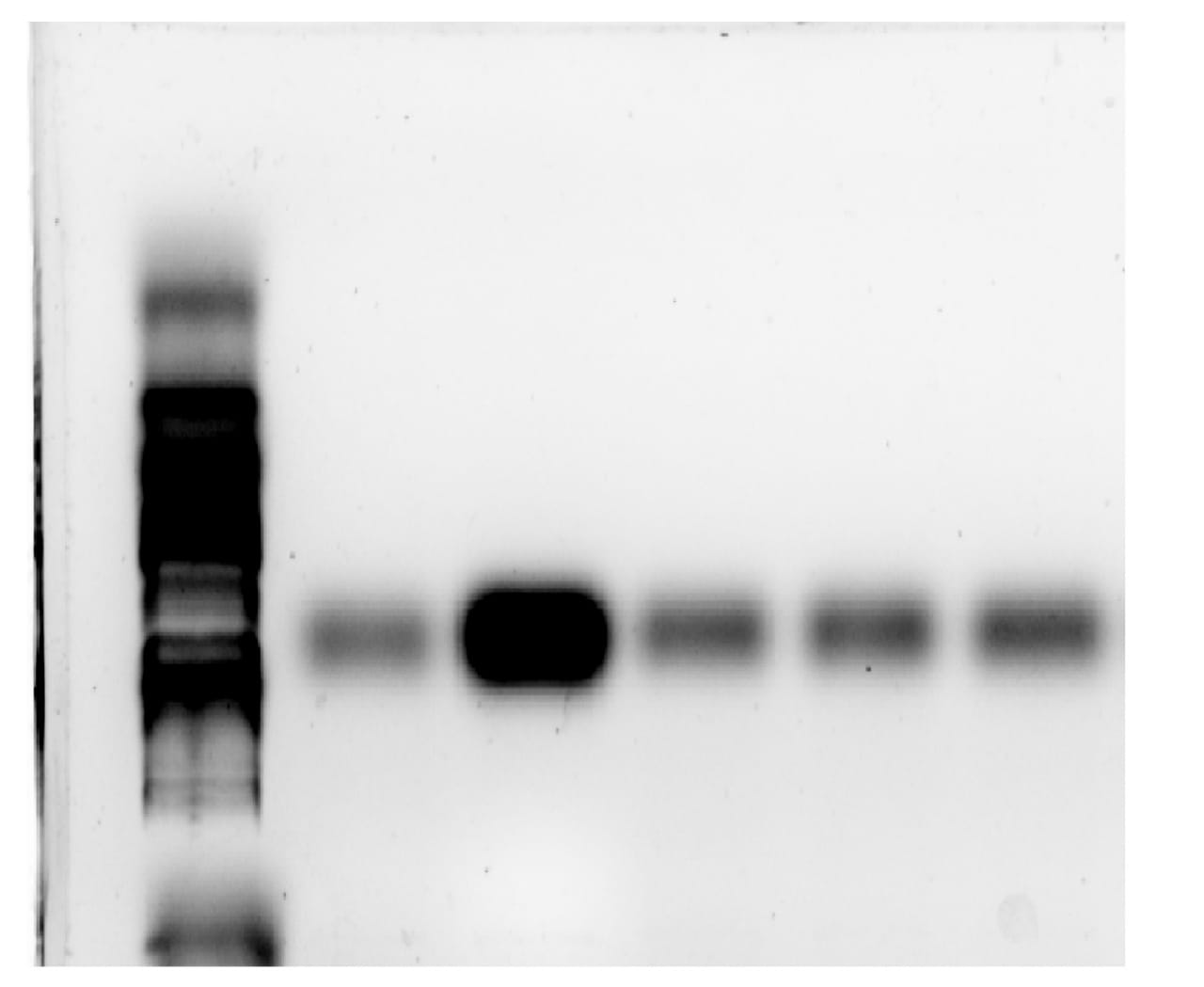

Supplement: S2 Fig — (JPG) [file pone.0220548.s002.jpg]

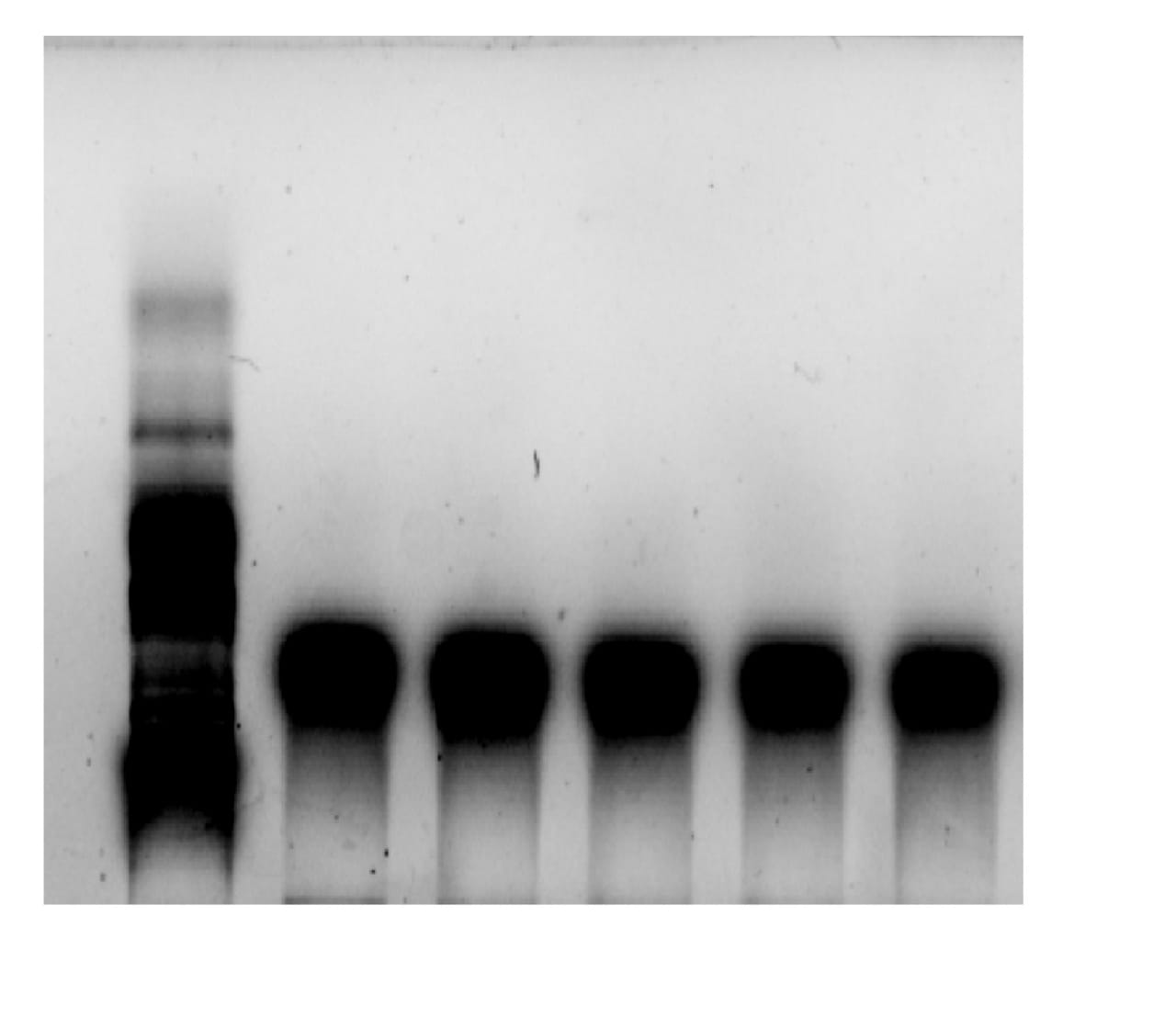

Supplement: S3 Fig — (JPG) [file pone.0220548.s003.jpg]
